# Effect of Polyvinyl Alcohol (PVA) Containing Artemether in Treatment of Cutaneous Leishmaniasis Caused by *Leishmania major* in BALB/c Mice

**DOI:** 10.5812/jjm.9696

**Published:** 2014-05-01

**Authors:** Parisa Ebrahimisadr, Fatemeh Ghaffarifar, Zuhir Mohammad Hassan, Mohammad Sirousazar, Fatemeh Mohammadnejad

**Affiliations:** 1 Department of Parasitology, School of Medical Sciences, Tarbiat Modares University, Tehran, IR Iran; 2 Department of Immunology, School of Medical Sciences, Tarbiat Modares University, Tehran, IR Iran; 3 Faculty of Chemical Engineering, Urmia University of Technology, Urmia, IR Iran

**Keywords:** *Leishmania major*, Artemether, Polyvinyl alcohol

## Abstract

**Background:**

Polyvinyl alcohol (PVA) is one of the well-known polymers, which has been used in numerous biomedical applications because of its good biocompatibility.

**Objectives:**

Due to problems made by the therapeutics already used for leishmaniasis, the aim of this study was to evaluate the effect of PVA containing artemether in treating cutaneous leishmaniasis in BALB/c mice.

**Materials and Methods:**

Aqueous solution of PVA was prepared by mixing with Double Distilled Water. After preparation of PVA, 4.33 mg of each drug (main drug artemether and control drug 14% glucantime) was added to 100 g of prepared PVA-honey solution. The solution was incubated at 37°C and the release of artemether was evaluated by measuring absorbance at 260 nm wave length. In this study for treatment of mice lesion, we used PVA containing artemether and glucantime and this method was compared with ointment treatment.

**Results:**

Mean diameters of lesions in mice treated with artemether were smaller than the control group and the differences were significant (P < 0.05). The mean lesion size of mice treated with PVA containing artemether in comparison with the group treated with ointment of artemether were smaller and the differences were significant (P < 0.05).

**Conclusions:**

PVA containing artemether is a new method for treatment of cutaneous leishmaniasis and according to the obtained results, artemether is an appropriate and effective drug, especially when used with PVA as a lesion dressing; thus we suggest that this method can be applied for the treatment of cutaneous leishmaniasis.

## 1. Background

Leishmania is a protozoan parasite which lives either as extracellular promastigotes in phlebotominae insects, or as intracellular amastigotes inside macrophages of mammalian hosts. Chemical drugs for treatment of leishmaniasis include pentavalent antimonate glucantime, pentostam, allopurinol and allopurinol riboside, amphotericin B, aromatic diamidianes, and paramomycin. Furthermore, physical methods such as curettage, surgical removal, ray therapy, heat therapy, and cryotherapy have been used (1, 2). Using these treatment methods lead to problems such as relapse, drug resistance, adverse drug reaction, secondary bacterial infection, and high costs of treatment (3-5). Therefore, a group of researchers have used insect products and medicinal plants, such as *Peschiera australis*, *Peschiera vanherokii*, *Altharea rosa*, *Altharea officinalis*, *Leguminosa faliacaea*, *Alkanna tinctoria*, *Pegamum harmala*, and *Euphorbia mysinites, as a means of treatment*. These plants have had inclusively positive results (4, 6-8).

Artemether as one of the semi-synthetic derivatives of artemisinin (artemisinin were discovered from Artemisia plant leaves) has the structural formula of C_16_H_26_O_5_ and molecular weight of 298.4. Artemisinin is structurally different from other well-known anti-malarial drugs and has a different mechanism of action. Scientists believe that artemisinin’s strong action against parasites is due to the presence of Endoperoxide Bridge. The suggested mechanism of action for artemisinin is based on the unusual chemical structure of the compound’s core, which participates in the formation of peroxide bridge and is also essential for its anti-malarial activity. Without an oxygen atom, artemisinin is inactive (9). Peroxides are well-known sources of reactive oxygen including hydroxyl and superoxide free radicals. Hence, it has been suggested that free radicals can play a role in artemisinin’s mechanism of action. Role of free radical in biological mechanism of action for artemisinin’s derivatives such as artemether was confirmed during the late 1980s (10).

We also have used polyvinyl alcohol (PVA) as a lesion dressing for greater drug delivery efficacy. PVA is a well-known polymer, which because of its good biocompatibility, has been used in numerous biomedical applications, for example as implants (11), artificial organs (12), contact lenses (13), drug delivery devices (14), and also lesion dressings for lesions management (15-17). PVA has certain advantages that make it an excellent candidate for biomaterials. Some of these advantages include its non toxic, non carcinogenic and bio-adhesive characteristics, as well as its associated ease of processing. PVA has an uncomplicated chemical structure and modifications are possible by simple chemical reactions. In addition PVA gels exhibit a high degree of swelling in water (or biological fluids) and a rubbery and elastic in nature. Due to these properties, PVA is capable of simulating natural tissues and can be readily accepted in the body.

## 2. Objectives

Due to problems made by the therapeutics already used for leishmaniasis, the aim of the present study was to evaluate the effect of PVA containing artemether in treating cutaneous leishmaniasis in BALB/c mice.

## 3. Materials and Methods

### 3.1. PVA Preparation

In all experiments we used commercial grade PVA with a polymerization degree of 1700 and a saponification value greater than 98%, which was purchased from the Nippon Synthetic Chemical Industry Co., Ltd, Japan. Double distilled water (DDW) was used to prepare all aqueous solutions.

### 3.2. Artemether Preparation

Artemether was obtained from Exim Pharm Co. US. Stock solutions of artemether were freshly prepared in ethanol-water (1:1, w/w). At first 1 mg of artemether was dissolved in 0.5 mL of ethanol and then slowly diluted with 0.5 mL of water. For better dissolving, we used 100 μL of ethanol and 100 μL of water to obtain a volume of 1 mL.

### 3.3. Optimization and Preparation of PVA Containing Artemether

We prepared four concentrations of artemether in DDW (5, 10, 25 and 50 μg/mL) and the absorbances were measured at 260 nm wave length. Next, a standard carve was plotted. A piece of PVA gel containing artemether (1 mg/mL) was placed in a container with 100 ml of DDW and incubated at 37˚C and the release of artemether was evaluated 5, 15 and 24 hours after incubation. The concentration of released artemether was measured by reading absorbance at 260 nm wave length. Next, the release curve was plotted according to the released artemether at different time points. The results of release assay showed that after 24 hours about 48% of artemether was released. According to this result, we determined the suitable concentration of artemether in PVA gel.

#### 3.3.1. Preparation of Transdermal and Injectable Systems of PVA

Aqueous solution of PVA was prepared by mixing in Double Distilled Water. The solution was mixed slowly and heated to 90°C for a period of about 4 hours to achieve complete dissolution. Then, honey (The properties of the honey included: water activity (aw) =0.6; pH = 4; acidity =22 meq/kg honey; sucrose = 7.5%; reducing sugars =75%; total sugars =82.5 % and humidity = 14%) with a 1:1 ratio (w/w) with respect to PVA was added to the solution to attain final PVA-honey viscous solution. Finally, the solution was cooled down and 4.33 mg of each drug (main drug artemether and control drug glucantime = 14%) was added to 100 g of prepared PVA-honey solution and mixed slowly. About 1.2 g of each sample (containing 52 of artemether and control drug glucantime) was used as a transdermal drug delivery system for each animal daily and then the diameter of the lesion was measured daily by a caliper for two weeks. About 1.2 g of each sample (containing 52 of artemether and control drug glucantime) was used as an injectable system, then diameter of the lesion was measured weekly by a caliper for three weeks.

#### 3.3.2. Preparation of Film and Implantable Systems of Gel

PVA-Honey solutions containing artemether as the main drug and glucantime as the control drug were prepared according to the method and ratios described in section 3.1 In order to prepare the solid PVA-honey hydrogel films, the prepared aqueous solutions were poured into flat plastic moulds (having thicknesses of 3 mm) and placed at -20˚C for 24 hours to induce crystallization (freezing step). After the freezing process, hydrogels were subsequently allowed to thaw for 24 hours at 23˚C (thawing step). This cyclic process was repeated three times for each solution. A piece of hydrogel (5 × 5 mm) with 1.2 g weight containing 52 of artemether and glucantime as the control drug was used as an implantable and thin film drug delivery system for each animal, then diameter of the lesion was measured weekly by a caliper for three weeks.

### 3.4. Ointment Preparation

250 µg of artemether was mixed in 1 mL of common cream including Glycerin and then 0.1 mL of ointment was rubbed on the lesion. Diameter of the lesion was measured daily by a caliper for two weeks.

### 3.5. Parasite Culture

*Leishmania major* (MRHO/IR/75/ER) was cultured in RPMI 1640  (Gibco-BRL, Life Technologies Ltd., Paisley, Scotland) with 20% FCS  (Gibco-BRL, Life Technologies Ltd., Paisley, Scotland). for preparation of adequate promastigotes.

### 3.6. BALB/c Contamination Method With *L. major*

BALB/c mice aged 6-8 weeks were purchased from the Razi Institute (Karaj, IR Iran) and were kept in the animal house under standard conditions of food and water. About 2 × 10^6^ promastigotes of *L. major* (MRHO/IR/75/ER) were injected to the base of the tails of mice in stationery phase. The same volume of PBS (for control) was injected into the left leg and do not observe the reaction. After three to five weeks, the wound was observed at the injection site of the parasite.

Mice were divided in to five categories:

1. Healthy control group, the group for which only PBS was injected, and no parasites were observed to have injury (five mice);
2. Infected control group without treatment (five mice);
3. Infected groups treated with PVA containing glucantime in local, injected, hydrogel and implant forms (five mice in each situation);
4. Infected groups treated with PVA containing artemether in local, injected, hydrogel and implant forms (five mice in each situation);
5. Ointment containing artemether (five mice).

### 3.7. Statistical Analysis

Results of each experiment were summarized with mean and standard deviation (SD) values. Mean lesion size was compared with Mann Whitney and Anova-one way tests (P values of < 0.05 were considered significant) by using SPSS version 16.

## 4. Results

### 4.1. Results of the Groups Under Study

In the healthy control group, all mice were healthy at the end of experiment. According to the results, all mice in the infected group, which were left untreated, eventually died at the end of the experiment. The mean diameter continuously increased thus in some mice metastasis resulted.

#### 4.1.1. Groups Treated With Artemether by Ointment Form

**Figure 1.**
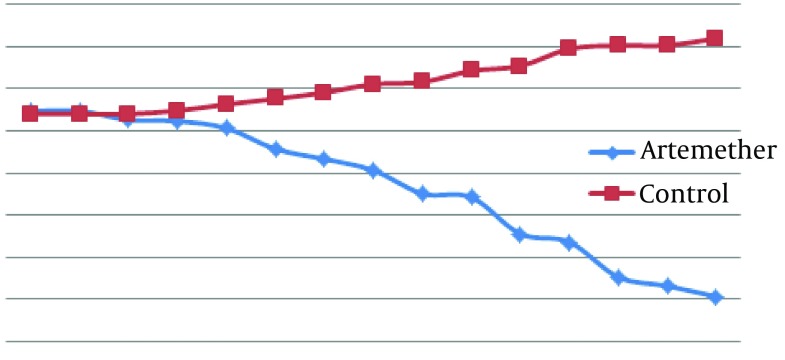
Comparison of Mean Lesion Diameter (cm) Caused by L. major in BALB/c Mice Untreated and Treated With Artemether With Ointment for 14 Days

According to Figure 1, mean diameter of lesion in the infected group treated with artemether was smaller than the untreated infected group and from five days after treatment the differences were significant between mean diameters of lesion in the two groups (P < 0.05).

#### 4.1.2. Groups Treated With Glucantime and Artemether by Local Form of Gel

**Figure 2.**
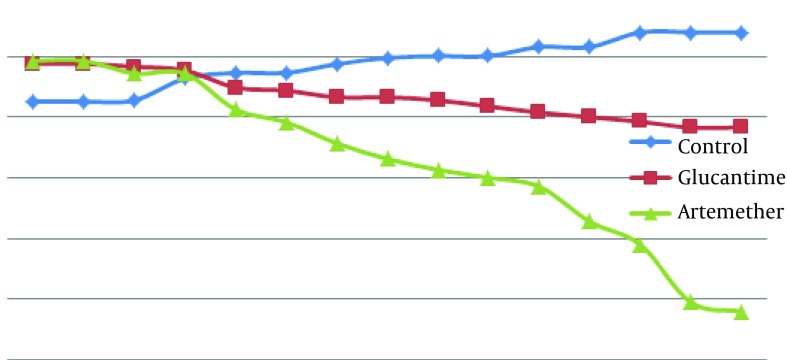
Comparison With Lesion Diameter (cm) Caused by *L. major* in BALB/c Mice in Treated and Control Groups Infected Group Infected group treated with glucantime and infected group treated with artemether by local form for 14 days.

According to Figure 2, there were no significant differences between mean diameter of lesion in the three groups at the first, second and third days after treatment (P > 0.05). There was a significant difference between the infected group treated with artemether in comparison with the untreated infected group, from the fourth day after treatment (P < 0.05). There was a significant difference between the infected group treated with artemether in comparison with the group treated with glucantime from the fifth day after treatment (P < 0.05).

#### 4.1.3. Groups Treated With Glucantime and Artemether by Injection Form of Gel

According to Table 1, in the first and second weeks after treatment there were no significant differences between mean diameter of lesion in the three groups (P > 0.05). On the third week after treatment, there was a significant difference between infected groups treated with artemether (Table 1) in comparison with the untreated infected group (P < 0.05).

**Table 1.** Comparing Changes in Lesion Diameter (cm) Caused by *L. major* in BALB/c Mice in the Treated and Untreated Infected Groups ^a^, ^b^

Week	Lesion Diameter, cm			
	Infected Groups Treated With Artemether	Infected Groups Treated With Glucantime	Untreated Infected Group
**0**	1.310 ± 0.118	1.71 ± 0.668	1.37 ± 0.553
**1**	1.175 ± 0.091	1.68 ± 0.707	1.43 ± 0.579
**2**	0.855 ± 0.205	1.65 ± 0.721	1.775 ± 0.657
**3**	0.3 ± 0.084	1.55 ± 0.650	1.93 ± 0.650

^a^ Data are presented in Mean ± SD.

^b^ Infected group treated with Glucantime, infected group treated with artemether by injected form of gel for three weeks.

#### 4.1.4. Groups Treated With Glucantime and Artemether by Implant Form of Gel 

According to Table 2, there were no significant differences between mean diameter of lesion in the three groups in the first and second weeks after treatment (P > 0.05). There was a significant difference between the infected group treated with artemether in comparison with the untreated infected group on the third week after treatment (P < 0.05).

**Table 2.** Comparing Changes in Lesion Diameter (cm) Caused by *L. major* in BALB/c Mice in the Untreated Infected Group ^a^, ^b^

Week	Lesion Diameter (cm)			
	Infected Groups Treated With Artemether	Infected Groups Treated With Glucantime	Untreated Infected Group
**0**	2.287 ± 0.594	2.199 ± 0.438	1.87 ± 0.141
**1**	2.133 ± 0.564	2.016 ± 0.652	2.21 ± 0.088
**2**	1.716 ± 0.605	1.866 ± 0.537	2.46 ± 0.270
**3**	1.62 ± 0.558	1.793 ± 0.472	2.53 ± 0.315

^a^ Data are presented as Mean ± SD.

^b^ Infected group treated with glucantime, infected group treated with artemether by implant form of gel for three weeks.

In the infected groups treated with hydrogel form of artemether, drug could not penetrate into the lesion due to yielded drought; hence this method cannot be used to recover the lesion.

#### 4.1.5. Groups Treated With Glucantime and Artemether by Hydrogel Form

For this form of PVA, which contains glucantime and artemether, we could not show any recovery in lesions of cutaneous leishmaniasis. There were no significant differences in lesion size of treated groups and control group during the entire time of treatment.

## 5. Discussion

In leishmaniasis, effective treatment can considerably reduce the social and mental consequences of this disease and assist for its control and prevention. The drugs of choice for treatment of cutaneous leishmaniasis are glucantime and Pentostam. Both of them have toxic side effects. Pharmaceutical research represents a major strategy for discovering new drugs with minimal toxicity. Adverse effects related to artemisinin derivatives are rare (18, 19). Artemether is effective for treatment of parasitic diseases including Schistosoma (20), Clonorchis (21), *Fasciola* (22), malaria (23), *L. infantum* (24) and *L. major* (25, 26). Similar to malaria parasite, the trematodes swallow the host’s hemoglobin as their food. Therefore, artemether can be activated by heme or other iron compounds through production of toxic compounds or iron free radicals (25). 

Artemether is an effective drug but its half life is short (three hours). Due to the short half-life of artemether, we used PVA for control of drug dosage in oral and injection forms. We chose PVA as it could absorb body fluids and act as a barrier against bacteria (17). The results show that the infected group treated with ointment containing artemether, means of lesion diameter decreased from 1.094 cm to 0.214 cm and in the infected group treated with PVA containing artemether, which was used in the local form, means of lesion diameter decreased from 0.986 cm to 0.16 cm; therefore in the infected group treated with PVA containing artemether recovery through decrease in lesion diameter was greater than the infected group treated with ointment containing artemether during two weeks. 

Comparison of the three groups including the infected group without treatment, the infected group treated with PVA containing artemether and glucantime for which implant and injected forms were used, showed that in the infected group treated with PVA containing artemether, the lesion size was smaller than the other groups during three weeks. The infected group treated with PVA containing artemether in hydrogel form, could not obtain lesion recovery; this may be related to the kind of lesion for cutaneous leishmaniasis caused by *L. major* that is dry in the mouse model, whereas hydrogels need wet lesions to release the drug. Among all these methods used in this study, the local form of PVA containing artemether due to its easy usage, not causing pain and shortening the lesion recovery period, was the best option. According to the obtained clinical results, it is a very promising drug which can be suggested as an effective, suitable, and even an alternative method for treatment of leishmaniasis.
